# Albuminaria

**Published:** 1890-07-12

**Authors:** 


### ALBUMINURIA.

We have several times referred to albuminuria and its relation to the important question of life insurance. We observed that, notwithstanding the occasional occurrence of functional albuminuria, the passage of albumen must be regarded with suspicion, especially as it has recently been argued that the passage of albumen creates that condition of kidney known as Bright's disease. Dr. Goodhart, physician to Guy's Hospital, has recently published two clinical lectures on albuminuria (B. M. J. May 24th), in the first of which he states that functional, physiological, or cyclic albuminuria is a condition much less common than has come to be accepted. With this we fully agree. Dr. Goodhart's rule of practice is that if a case presents itself for examination and albumen be found in the urine it is a one for further investigation. If the albumen is persistent, it must be regarded as a danger signal. Dr. Goodhart considers that the terms "functional" and "physiological" have little warrant when applied to albuminuria, and ought to be discarded, but that "intermittent" might be retained.

Dr. Goodhart's second lecture is headed "The vagaries of renal disease." First serious kidney disease may not be accompanied by the ordinary symptoms. Several cases of the kind are given. Another feature which may be called a vagary is intermission in the passage of albumen. A case of nephritis may run on for months with urine now containing much albumen, and then for several days scarcely a trace. Another vagary is the rapid recovery of cases that seemed beyond hope. Cases occur which from the severity of the albuminuria and the extent of the dropsy, do not appear likely to live, but who survive as chronic invalids for years. The most marked instances of rapid recovery are, however, in children. Another deviation from the ordinary course of nephritis is the occasional substitution of a state of low tension for high. The ordinary pulse of renal disease is a hard one, or one of high tension. But this does not occur in some cases of nephritis, and when low tension exists the case is likely to do badly.

